# Insights into the Mechanisms Involved in Lead (Pb) Tolerance in Invasive Plants—The Current Status of Understanding

**DOI:** 10.3390/plants12112084

**Published:** 2023-05-24

**Authors:** Muhammad Rahil Afzal, Misbah Naz, Justin Wan, Zhicong Dai, Raza Ullah, Shafiq ur Rehman, Daolin Du

**Affiliations:** 1 Institute of Environment and Ecology, School of Environment and Safety Engineering, Jiangsu University, Zhenjiang 212013, China; misbahnaz.ray@yahoo.com (M.N.); 1000005143@ujs.edu.cn (J.W.); daizhicong@163.com (Z.D.); 2 Institute of Environmental and Agricultural Science, Faculty of Life Sciences, University of Okara, Okara 56130, Pakistan; raza1838@gmail.com; 3 Department of Botany, Faculty of Life Sciences, University of Okara, Okara 56130, Pakistan; evergreenpk@gmail.com

**Keywords:** invasive plant species, ecosystem services, lead (Pb), mechanisms of tolerance, contaminated environments

## Abstract

Invasive plant species possess remarkable abilities to establish themselves in new environments and to displace native species. Their success can be attributed to various physiological and biochemical mechanisms, allowing them to tolerate adverse environmental conditions, including high lead (Pb) toxicity. Comprehension of the mechanisms responsible for Pb tolerance in invasive plants is still limited, but it is rapidly evolving. Researchers have identified several strategies in invasive plants to tolerate high levels of Pb. This review provides an overview of the current understanding of the ability of invasive species to tolerate or even accumulate Pb in plant tissues, including vacuoles and cell walls, as well as how rhizosphere biota (bacteria and mycorrhizal fungi) help them to enhance Pb tolerance in polluted soils. Furthermore, the article highlights the physiological and molecular mechanisms regulating plant responses to Pb stress. The potential applications of these mechanisms in developing strategies for remediating Pb-contaminated soils are also discussed. Specifically, this review article provides a comprehensive understanding of the current status of research on the mechanisms involved in Pb tolerance in invasive plants. The information presented in this article may be useful in developing effective strategies for managing Pb-contaminated soils, as well as for developing more resilient crops in the face of environmental stressors.

## 1. Introduction

Soil-heavy metal pollution and biological invasion are two major environmental concerns that have the potential to negatively impact agricultural output, ecosystem health, and biodiversity on a global scale [1,2]. Heavy metals are known to inhibit plant growth and productivity; on the other hand, they can also provide an opportunity for plants with high tolerance to thrive in problem soils [3,4,5]. Many invasive species have been found to successfully colonize heavily metal-polluted areas due to their ability to exploit diverse physiological niches [6,7,8]. It is vital to investigate the mechanisms of heavy metal tolerance and competition among interacting species in response to heavy metal pollution.

Heavy metals, including lead (Pb), can negatively impact photosynthetic activity and cause biomass and fitness loss in plants by interfering with physiological and biochemical responses, such as photosynthesis [9,10,11]. To combat this, plants have developed adaptive strategies to prevent metal uptake in photosynthetically active tissues and to activate cellular detoxification mechanisms [12,13,14]. Metllophytes and hyperaccumulators can absorb and store heavy metals, particularly in their above-ground parts, such as leaves, at levels that surpass specific metal thresholds [15]. According to previous studies, the concentrations of the heavy metals in hyperaccumulators can be 10–500 times higher than in other plants without causing any adverse effects [16,17]. However, the uptake of metals by the plant is influenced by various factors, such as soil pH, organic matter, the type of metal, and the presence of other soil ions (which may have antagonistic effects) [18,19]. Interestingly, some heavy metal-tolerant plants may exhibit metal deficiency symptoms when specific metals are absent from the soil. At low doses, these metals can even stimulate photosynthetic activity and pigment synthesis [20,21,22]. In this way, heavy metal-tolerant plants, including some invasive plants, may have a competitive advantage in habitats with low-level heavy metal contamination, such as Pb, which can facilitate their colonization.

The interaction of physiological processes, ecological functions, and genetic makeup within a range of environments may help plants to devise appropriate response strategies following anthropogenic ecosystem modification [23]. Numerous invasive plants have been found to possess stronger ability to endure or accumulate Pb in their tissues, providing them with a competitive edge over native plant species in inter-specific competition [24,25,26]. This is due to the inherent advantages of invasive plants in terms of growth, fecundity, and physiological properties, such as phenotypic plasticity and allelopathy [27,28,29]. In polluted sites, some invasive plants have been observed to grow more prominently than neighbouring native plants, indicating that they may have changed the species' composition. Invasive plants can also harm native plants through plant–soil feedback effects, whereby they rapidly adapt to the invaded environment and change the heavy metal availability in the soil [30,31,32]. Therefore, the competitive performance difference among alien and native plant species under heavy metal Pb stress can directly impact the ecological balance in polluted areas. However, it is important to note that the ability to tolerate high Pb levels is not a universal characteristic of all invasive species, and it may vary depending on the specific species and environmental conditions [33].

### 1.1. Biogeochemistry of Pb in Different Soils

Pb is a naturally occurring element in soil, but human activities have significantly increased its concentrations in the environment, leading to potential health hazards for humans and the ecosystem. The biogeochemistry of Pb in the soil is influenced by various factors, including soil type, pH, organic matter content, and the presence of other contaminants. 

Soil pH is an essential factor that affects the biogeochemistry of Pb in soil. The solubility of Pb increases in the pH range of 5.5 to 7.0. In acidic soils, Pb forms insoluble compounds, reducing its bioavailability to plants and other organisms [34,35,36]. In contrast, Pb may react with other compounds to form insoluble precipitates in alkaline soils [37,38]. Organic matter content in the soil is another crucial factor affecting the biogeochemistry of Pb. Organic matter can bind Pb by creating stable complexes, such as metal–humus in the soil, making it less bioavailable to plants and other organisms. However, soil organic matter can also increase the availability of Pb to plants by improving its solubility, increasing the cation exchange capacity (CEC) of soil and supplying metal chelates in the soil solution [39,40]. Soil texture, specifically the amount of surface area available for adsorption and desorption of Pb, can also influence the biogeochemistry of Pb. Fine-textured soils, such as clay, have a higher surface area than coarse-textured soils, such as sand, providing more adsorption sites for Pb [35]. However, fine-textured soils also tend to hold Pb more tightly, reducing its bioavailability to plants and other organisms [41,42]. The presence of other contaminants in soil can also affect the biogeochemistry of Pb. For example, the presence of cadmium, zinc, or copper can compete with Pb for binding sites on soil particles [43]. Furthermore, the presence of organic pollutants, such as polycyclic aromatic hydrocarbons (PAHs), can increase the availability of Pb by disrupting soil organic matter [44,45].

### 1.2. Plant Invasion and Pb Dynamics in Soils

**Figure 1:**
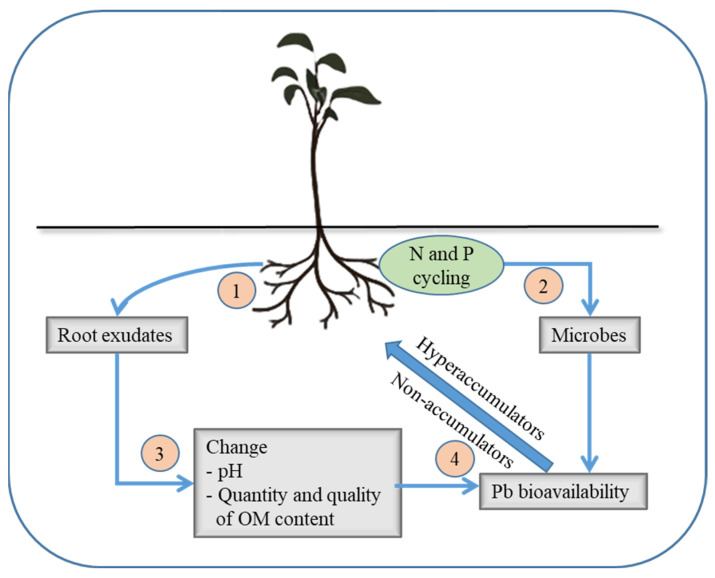
The interaction between plant invasion and Pb dynamics in polluted soils.

Plant invasion can play a significant role in regulating biogeochemical cycles of heavy metals, including Pb in the invaded ecosystems that may generally include limiting (stabilization/immobilization) or enhancing (mineralization) the bioavailability of the heavy metal (Figure 1). The impacts of invasive plants on Pb dynamics in the soils are complex and depend on the specific species and ecosystem context. Invasive plant species can increase the accumulation of Pb in soil. A study by Guo, et al. [46] found that the invasive plant species *Ageratina adenophora* had higher Pb accumulation in its tissues compared to native plant species in Pb-contaminated soil, and it increased the total Pb content in soil due to its high biomass production and litter decomposition. Invasive plant species can change soil pH, as well as the quantity and quality of organic matter content through their litter production and root exudates, which can impact the binding of Pb to soil particles and affect its bioavailability to plants and other organisms [47,48,49]. A study by Gajaje, et al. [50] found that the invasive *Nicotiana glauca* lowered pH and increased organic matter content of the rhizosphere soil compared to the bulk soil, which increased the solubility of Pb in soil and, ultimately, the accumulation of this metal into the root and shoot tissues of this species. The study suggested that the invasive species may help to mobilize Pb in the soil and increase its bioavailability for hyperaccumulators. Invasive plant species can alter the soil microbial community, which may be attributed to the enhanced availability of nitrogen and phosphorus by regulating the cycling of these nutrients [51,52,53,54], and this, in turn, can affect the biogeochemical cycling of Pb. A study by Batten, et al. [55] found that the invasive plant species, *Centaurea solstitialis* (yellow starthistle) and *Aegilops triuncialis* (barb goatgrass), in comparison to five native species, showed an increased abundance and diversity of soil microbial communities, which enhanced the transformation of Pb into its less toxic form in the soil (Table 1). This study suggested that the invasive species may have a positive impact on soil microbial activity and Pb biogeochemistry.

Taken together, these studies suggest that plant invasion can have complex and varied effects on Pb dynamics in soils. Invasive species may increase Pb accumulation in the soil through their high biomass production, but they may also help to immobilize Pb through changes in soil properties and microbial activity. More research is needed to understand the mechanisms underlying the impacts of plant invasion on Pb dynamics in soils and how these impacts may affect soil quality and ecosystem functioning.

## 2. Mechanisms Involved in Invasive Plants’ Tolerance to Pb

**Figure 2:**
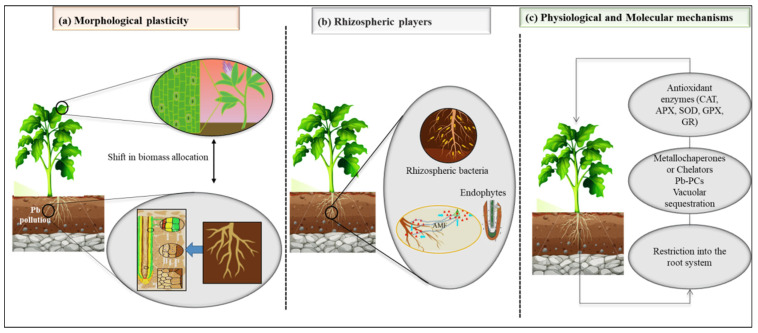
The mechanisms of invasive plants’ adaptation to Pb-polluted environments.

Invasive plant species have been known to possess the ability to tolerate and adapt to a variety of environmental stresses, including heavy metal pollution. Pb pollution is a widespread problem that can negatively impact plant growth and development. However, some invasive plant species have been shown to possess a higher degree of Pb tolerance than native plant species [9,57]. Understanding the mechanisms underlying Pb tolerance in invasive plants can provide insights into the strategies that invasive plants use to cope with environmental stress (Figure 2) (Table 1).

### 2.1. Morphological Plasticity

As an adaptive strategy, invasive plants create higher morphological shifts and biomass plasticity to combat the negative impacts of high environmental levels of heavy metals in inter-specific competition with neighboring native plant species [24,26,58], which may contribute to reduce the metabolic costs of metal detoxification [59]. For instance, some plants maintain a relatively higher root-to-shoot ratio in polluted soils, which helps enhance their capacity to store noxious metallic ions and facilitates the homeostasis of various cellular biomolecules in the roots involved in plant defense-related mechanisms [9,60]. This comparative shift in biomass distribution from shoots to roots may impose an increase in the requirement for nutrients, as well as other limiting resources as a consequence of local nutrient imbalances in polluted soils. However, there have been reports of decreased biomass allocation, which can be attributed to heavy metal concentrations in the soil and the growth strategies adopted by different plant species. This can lead to lower absorption or avoidance of pollutant uptake by roots [60,61,62]. In fact, Yang, et al. [63] found that invasive species, such as *Biden frondosa*, *Erigeron Canadensis*, *Alternanthera philoxeroides* and *Cynodon dactylon*, accumulated significantly lower levels of Pb in both roots and shoots compared to native species. They suggested that these species employed an extrusion strategy to tolerate the heavy metal. Metal excluders are preferred for stabilizing metals in contaminated areas, as they have a low translocation rate to above-ground tissue. This minimizes the likelihood of heavy metals entering the food chain and, thus, reduces the risk of adverse effects on the ecosystem [64,65]. In some cases, plant morphology is not affected by the presence of higher levels of heavy metals. For example, there was no negative effect of even a high dose of Pb found on the morphological characteristics of a *Fallopia x bohemica* hybrid [66]. Moreover, plant height in Japanese Knotweed s.l. was even significantly greater compared to the control [67], showing that the toxic metals, depending upon the level of pollution, had a triggering effect on the growth of these invasive species. 

### 2.2. Contribution of Rhizospheric Biota

Heavy metal toxicity impacts both the plants and the microorganisms in polluted soils [68,69,70]. However, invasive plant species have unique characteristics that enable them to combat this major ecological factor. Invasive plants can significantly alter the abundance and functional diversity of microbial communities, which are resistant and tolerant to heavy metals and develop strong plant–microbe interactions, resulting in positive feedback [55,71]. This may give them a competitive advantage that can affect the fitness of native plants and ecosystem function in contaminated environments. According to Marschner and Timonen [69], the microbial communities in the rhizosphere of various plant species that are growing in the same soil are typically unique. Similarly, Li, et al. [72] found that the bacterial communities in the rhizosphere of mine tailings were different for the invasive plants *Erigeron annuus, Imperata cylindrical,* and *Pueraria lobata*, as well as the native plant species *Lysimachia clethroides* and *Equisetum ramosissimum*. It was suggested that this variability may be attributed to the varying composition of root exudates and litter, which differed across the different plant species. The adaptive and evolutionary capabilities of the rhizospheric biota in invasive plants may facilitate their successful colonization and survival in mine tailings [73], which could involve a variety of mechanisms, including metal immobilization, chelation, and detoxification.

Invasive plant species have been reported to recruit beneficial microbes, including endophytes and rhizospheric bacteria and fungi, to promote their growth and tolerate environmental stresses, including heavy metal stress [74,75]. For example, the annual invasive plant *Erigeron annuus* was found to thrive in lead–zinc tailings, with its rhizosphere exhibiting higher bacterial numbers, urease and β-D-glucosidase activity, and structural diversity indices compared to the native species *Lysimachia clethroides*, resulting in a reduced bioavailability and ultimately promoting phytostabilization of Pb in the rhizosphere [72]. Microbiota also colonize the phyllosphere and endophytic compartments within plants. Some plant growth-promoting bacteria (PGPR) have been recognized as endophytic microbes and can help the host plant tolerate stressful environments, including heavy metal toxicity by promoting plant growth and protection [76,77]. Invasive *Senecio vulgaris* is a Pb-tolerant plant species often found in heavy metal-contaminated soils [78], and it was reported to host a high abundance of heavy metal-resistant, phosphate-solubilizing, and nitrogen-fixing bacteria, including *B. diminuta* and *R. leguminosarum* in its roots and leaves, which might be responsible for the adaptation of this species to metal stress in a nutrient poor soil environment [77]. Evaluating the ecology of such bacteria in association with their host plant can be important in understanding more about the mechanisms of plant tolerance to heavy metals in polluted soils. Moreover, the impacts of the structural distribution and the dynamics of the rhizospheric and endophytic bacteria on exotic plant invasion are species-specific and may vary according to the heavy metal concentration in a particular environment [79,80].

Arbuscular mycorrhizal fungi (AMF) are among the most important soil microbes that are involved in forming a mutualistic association with most terrestrial plants [81], including alien plants [82], and they offer an interactive interface between plant roots and soil to stimulate plant growth, assist nutrient uptake, and enhance tolerance to adverse environmental conditions, such as heavy metal stress as a feedback mechanism [83,84,85,86]. It was found that the belowground AMF community composition played a significant role in facilitating the invasion process of *Biden pilosa* in comparison to native plant species [87]. Ruyi, Guodong, Jianjun and Xin [8] found that most of the Pb was sequestered in the rooting zone, i.e., roots and rhizome; moreover, it was suggested that higher efficiency of the mycorrhizae on nitrogen and phosphorus uptake might be responsible for the successful invasion of *Solidago canadensis* over native species in the soils polluted with Pb. 

Overall, the contribution of rhizospheric biota to the tolerance of invasive plants to Pb is significant and multifaceted. More research is required to gain a comprehensive understanding of the interplay between invasive plants, microorganisms, and rhizosphere soil. Moreover, it is vital to devise tactics that can disrupt the mechanisms through which these microorganisms assist invasive plants in thriving in soils contaminated with heavy metals. This is necessary to address the potential hazard posed by invasive plants to ecosystems. 

### 2.3. Physiological and Molecular Mechanisms

Physiological and biochemical adaptations play a critical role in determining the bioavailability of metals in plants [88,89]. Effective persistence and establishment of perennial invasive plants may depend on competitive and tolerance strategies [90,91]. In addition to competitive ability, higher or equivalent tolerance to stressful conditions in comparison to native species may lead to the successful invasion of plants. Physiological traits related to stress conditions, such as heavy metal toxicity, can directly impact fitness, and variation in these traits among exotic and native plant species may indicate a strategy for competition and invasive ability. For instance, maximizing photosynthesis can increase growth rates, production, and biomass accumulation, leading to increased competitive success [92].

Restricting metals in the root system exhibits a strategy to avoid or tolerate detrimental effects of Pb in the polluted soil environment. Generally, plants show a relatively low mobility of Pb. Therefore, most of Pb absorbed by the plant is retained in its roots [93], which are documented in various invasive species, including *Festuca rubra* [94] and *Brassica. juncea* [95]. It has been reported that Pb uptake in *Brassica. juncea* was significant and mainly intracellular, whereas there was a minimal uptake, occurring mainly extracellularly, located at a distance from the root tips [95]. Pb detection using histochemical methods revealed its substantial accumulation on the root surface, followed by pericycle cell walls of *Dianthus carthusianorum* [96]. Moreover, the invasive weed *Amaranthus spinosus* L. showed a restricted upward transportation of Pb from roots to shoots and an enhanced chlorophyll pigment profile in the leaves after exposure to the heavy metal, indicative of one of the tolerance mechanisms of the plant species [97]. Various cation transporters in the plasma membrane (PM) can serve as possible entry routes for toxic metals into plant cells. Because Pb is a nonessential plant element and is extremely noxious, it is unlikely that plant cells have specialized transporters for Pb [98]. A better comprehension of the underlying mechanisms of Pb uptake, translocation, and accumulation by the invasive plants is required for the effective utilization of Pb-tolerant plants in polluted soils. Researchers discovered a chain of membrane protein channels (NtCBP4, calmodulin-binding protein) in *Nicotiana tabacum* that transports Pb ions across the PM into the plant cell through calcium ion channels. This protein is similar to the ring nucleotide non-selective cationic channel protein of mammals, and it is the first known plant protein that can regulate the accumulation and tolerance of Pb in plants [98].

Once having breached the biophysical barriers, heavy metals infiltrate tissues and cells, triggering various mechanisms of cellular defense to counteract their detrimental effects. To endure or nullify metal toxicity, plants rely on diverse cellular biomolecule biosyntheses, including low-molecular-weight protein metallochaperones or chelators, such as putrescine, nicotianamine, mugineic acids, spermine, glutathione, organic acids, phytochelatins, and metallothioneins. Additionally, plants produce cellular exudates, such as flavonoids, heat shock proteins, phenolic compounds, specific amino acids (e.g., histidine and proline), protons, and hormones (e.g., salicylic acid, ethylene, and jasmonic acid), which further aid in metal detoxification [99,100]. Phytochelatins (PCs) have been reported to be utilized as biomarkers to detect heavy metal stress in plants at an early stage [101]. The presence of Pb chelated by thiol groups, possibly in the form of phytochelatins, has also been reported [102]. Andra, et al. [103] reported that Vetiver grass (*Vetiveria zizanioides*) can synthesize PCs and produce Pb–PCs complexes, and ultimately accumulate up to 19,800 and 3350 mg kg^−1^ DW Pb in root and shoot tissues, respectively. Pb detoxification in coontail (*Ceratophyllum demersum* L.) involves PC induction and the antioxidant system. PCs are produced in the cytosol and transported as high molecular weight metal–phytochelatin complexes to their final destination, the vacuole [14,104,105]. This compartmentalization of heavy metals has been proposed as an important detoxification mechanism in plants, including invasive plants. In the case of Pb, it involves up to 96% sequestration and sinking of the metal within plants parts, including the intercellular space, cell wall, vacuoles, and, partly, in the endoplasmic reticulum and dictyosome vesicles, which leads to a decrease in its toxicity in the cytosol and translocation to sensitive organs, such as leaves and seeds [106]. Phytochelatins produce stable Pb–PC complexes, which helps them, in vivo, to detoxify and to deposit heavy metal Pb [107,108]. PC retains soluble Pb inside the cytoplasm before its transport to vacuoles and chloroplasts, which minimizes the Pb^2+^ toxicity effect in the cells [109]. This process is governed by the metal uptake across the PM, facilitated by transport systems and intracellular high-affinity metal-binding groups. The negative membrane potential of the PM imparts the secondary transporters, including channel proteins and H^+^-coupled carrier proteins a strong driving force that induces the transport of metal ions [110]. Benaroya, et al. [111] found that Pb was stored in the cell wall and vacuoles of leaves of *Azolla filiculoides,* as revealed through TEM analysis. *Azolla filiculoides* plants exposed to Pb exhibited increased V-H^+^-ATPase activity, but lower protein content, compared to untreated plants, suggesting the involvement of tonoplasts in regulating the Pb accumulation secondary ion transporters in the vacuoles through the activity of H^+^-ATPase [111]. Further research on this mechanism of Pb tolerance in individual invasive plants, particularly in comparison to native species, needs serious attention. 

If these strategies fail to control metal toxicity, the balance of plant cellular redox systems is disrupted, resulting in higher production of reactive oxygen species (ROS) and, ultimately, oxidative stress [112]. This oxidative stress caused by heavy metals, including Pb in plants, may damage the cell membranes, inhibit the enzyme activity, and disrupt the metabolic processes. Plants employ enzymatic and non-enzymatic methods to combat the heavy metal-induced oxidative stress effects [113]. When exposed to higher metal concentrations, plants change gene expression by activating their antioxidative enzymatic machinery and produce enzymes, such as catalase (CAT), ascorbate peroxidase (APX), superoxide dismutase (SOD), guaiacol peroxidase (GPX), and glutathione reductase (GR), to help remove the free radicals formed as a result of oxidative stress due to the presence of heavy metals [114,115].

Moreover, non-enzymatic compounds, including phenolics, terpenes, flavonoids, alkaloids, and prolines, are also increased under metal stress and function as free radical scavengers [116]. This physiological response to heavy metal pollution varies among plant species; and, sometimes, changes depend on different plant organs, such as leaves, stems, and roots. Shukla, et al. [117] found that *Solanum viarum* (Dunal), along with its glandular trichomes (GTs), displayed higher antioxidant, ROS scavenging, and improved photosynthetic properties, as well as better cell wall stability when exposed to Pb, Cd, and Zn stresses, aiding in adaptation under heavy metal stress environment. GTs were found to be involved in heavy metals accumulation, detoxification, and excretion. *Solanum viarum* could be a higher biomass producing plant under heavy metal stress, and increasing GT density may improve HM accumulation and detoxification. Pb concentrations over 30 ppm are typically harmful to most plants [56].

However, Beals, et al. [118] demonstrated that *Alternanthera philoxiroides* can endure Pb concentrations of up to 50 ppm without showing any antioxidant peroxidase reaction. Unlike *Nasturtium officinale*, which demonstrated visible signs of stress, such as wilting and chlorosis when exposed to Pb at concentrations higher than 50 ppm, *Alternanthera philoxiroides* exhibited no apparent signs of stress [118].

Several studies have investigated the potential implications of genetic engineering to enhance the phytoremediation of Pb-contaminated soil systems. One approach involves the overexpression of genes that facilitate the transport and compartmentalization of Pb and other heavy metals within plant cells. For example, overexpression of the yeast cadmium factor *YCF1* in *Arabidopsis* and *Brassica juncea* plants led to improved Pb tolerance and its accumulation in vacuoles [119]. Similarly, overexpression of the wheat (*Triticum aestivum*) gene that encodes phytochelatin synthase (*TaPCS1*) in *Nicotiana glauca* (R. Graham) (shrub tobacco) plants enhanced their tolerance to Pb and resulted in increased biomass and Pb sequestration in mining soil [120].

In addition to *YCF1* and *TaPCS1*, other genes have also been found to play a role in Pb detoxification and resistance in invasive plants. For example, overexpression of *AtATM3* (a member of the ATP-binding cassette transporter family of *Arabidopsis thaliana*) in *B. juncea* led to improved tolerance to Pb^2+^ toxicity and higher Pb accumulation in shoot tissues. The higher capacity of *AtATM3* transgenic plants of *B. juncea* to tolerate and accumulate heavy metals could reflect the increased expression levels of *BjGSHII* (*B. juncea* glutathione synthetase II) and *BjPCS1* (phytochelatin synthase1), which themselves are induced by *AtATM3* overexpression [121]. In plants, *AtHMA3* would seem to have a similar function to that of *YCF1* in *Saccharomyces cerevisiae*, which also participates in vacuolar compartmentalization of noxious metals, including Cd and Pb [119,122]. Overall, genetic engineering shows promise for promoting the phytoremediation of Pb-contaminated soils, and future studies may identify additional genes and pathways involved in Pb detoxification and resistance in invasive plants.

## 3. Potential Applications

There are several potential applications of the mechanisms involved in Pb tolerance in invasive plants for developing strategies for remediating Pb-contaminated soils. Some primary methods and techniques employed to determine Pb tolerance in plants species are given in Table 2. By identifying and utilizing the mechanisms responsible for Pb tolerance, it may be possible to develop plant-based strategies for remediation of Pb-contaminated soils. One such application is phytoremediation, which is a green technology and a cost-effective approach that involves using plants to remove pollutants from contaminated soil [123]. Invasive plants, which evolved high Pb tolerance mechanisms, could be used in phytoremediation projects to remove Pb from the soil.

For example, the invasive plant *Brassica juncea* is highly effective at accumulating Pb in its tissues and has been used in several successful phytoremediation projects [124,125]. Other invasive plants, such as *Robinia pseudoacacia* and *Solidago canadensis*, have also been found to have high Pb tolerance and may have the potential for use in phytoremediation [126,127]. These plants are able to remove large amounts of Pb from contaminated soils, making phytoremediation an effective strategy for remediating Pb-contaminated sites. Researchers have successfully engineered plants with increased levels of transporters and phytochelatins, resulting in increased Pb tolerance [119,120]. These transgenic plants have the potential to be used for phytoremediation and for improving crop productivity in Pb-contaminated soils. However, the selection and management of plant species is an important part of the phytoremediation process. For example, using invasive plants for phytoremediation may pose risks, such as altering ecosystem processes and decreasing biodiversity if they escape and spread beyond their intended location [7]. Moreover, they may have different nutrient requirements and growth rates, which may affect their performance in different environmental conditions and limit their large-scale applications [128]. In some cases, using native plant species or non-invasive exotic species may be a more suitable alternative for phytoremediation [128]. Managing the remediation plants accumulating Pb is another challenge. There are several options for the disposal of harvested plants, including incineration of the plants and disposing of the plants in a hazardous waste landfill. However, the choice of disposal method may depend on various factors, such as the type and amount of heavy metals accumulated by the plants, the local regulations and guidelines, and the environmental and health risks associated with different disposal methods [129].

Another potential application of the mechanisms involved in Pb tolerance in invasive plants is in the development of Pb-resistant crops [130]. It has been reported that Pb-resistant maize varieties accumulated the lowest concentrations of Pb in kernels compared to other plant tissues [131]. By identifying and understanding the mechanisms that allow invasive plants to tolerate high levels of Pb, researchers could potentially develop crops that are better able to grow in Pb-contaminated soils. This could be especially important in areas where Pb contamination is widespread and where food security is a concern.

Finally, the mechanisms involved in Pb tolerance in invasive plants could also have applications in bioremediation. Bioremediation involves using microorganisms to break down pollutants in soil, water, or other environments [132]. Invasive plants that can tolerate high levels of Pb may have associated microorganisms that play a role in their ability to tolerate Pb [75]. Understanding these microorganisms and their interactions with invasive plants could potentially lead to the development of more effective bioremediation strategies for Pb-contaminated environments.

Taken together, the mechanisms involved in Pb tolerance in invasive plants have potential applications in developing strategies for remediating Pb-contaminated soils. By identifying and utilizing these mechanisms, it may be possible to develop plant-based phytoremediation strategies that are effective, sustainable, and eco-friendly. However, more research is needed to fully understand the complex interactions between plants, microorganisms, and heavy metals in soil systems to develop effective phytoremediation strategies.

## 4. Conclusions

Invasive plant species have developed mechanisms, such as tolerance and increased uptake and accumulation of Pb, production of detoxifying enzymes, and phytochelatins, to thrive in Pb-polluted environments. However, the current understanding of the mechanisms involved in Pb tolerance in invasive plants is still limited, and more research is needed to fully understand these mechanisms, especially in comparison to the native plant species.

## 5. Future Prospects

Given the adverse effects of Pb contamination on plant growth and development, the identification of the mechanisms involved in Pb tolerance in invasive plants has important implications for plant management and environmental remediation. Further research is needed to fully understand these mechanisms and their regulatory pathways. Additionally, the development of molecular tools to manipulate these mechanisms in plants could lead to the creation of Pb-tolerant crops that could be used to reduce the toxic effects of Pb in the environment. The exploration of natural variation in Pb tolerance among invasive plant species could also provide insights into the evolution of heavy metal tolerance mechanisms. Overall, the continued investigation of the mechanisms involved in Pb tolerance in invasive plants has the potential to provide valuable information for the management of invasive species and the remediation of contaminated environments.

## Figures and Tables

**Table 1:** Summary of different mechanisms of Pb tolerance observed in some invasive plant species.

Invasive Species	Growth Conditions	Location	Mechanism of Tolerance	References
*Solidago canadensis* L.	A greenhouse experiment with Pb as Pb(AC)_2_·3H_2_O @ (0, 300 and 600) mg kg^−1^ soil	Southeastern China	Exclusion or reduction in the uptake of Pb	[5]
*Solidago canadensis* L.	A greenhouse experiment with Pb as Pb(AC)_2_·2H_2_O @ (0, 300 and 600) mg kg^−1^ soil and Mycorrhizal inoculum	Southeastern China	High efficiency nutrient uptake in association with *mycorrhizae*	[8]
*Atriplex halimus*	Field study at 14 different sites, representing Pb pollution	Alexandria, Egypt	Molecular and physiological strategies involving transcriptional factors, ATPase transporter expression, and ROS detoxification	[23]
*Spartina alterniflora*	Greenhouse study with Pb-added sediment at (29, 68) µg/g Pb	Tuckerton, NJ, United States	Pb accumulator in above ground biomass (leaves and stems)	[11]
*Tetraena qataranse*	Pot experiment with Pb as (PbCl_2_) at (25, 50, and 100) mg/L Pb	Doha-Qatar	Pb hyperaccumulator With Increased antioxidant enzyme activity	[56]
* Lythrum salicaria *	Pot experiment with Pb at control (0, 1000 and 2000) ppm Pb	Cleveland, United States	Hyperaccumulator with death and regrowth strategy	[57]
*Centella asiatica*	Greenhouse experiment with Pb as Pb(NO_3_)_2_ at (0.20, 0.40 and 0.60) mg/L	Malaysia	Enhanced antioxidant capacity	[25]
*Conium maculatum*	two seasons of field surveys of Pb polluted area with 92 mg/kg dry soil Pb	Cook County, Illinois	Pb Accumulator	[6]

**Table 2:** Primary methods/techniques to determine Pb tolerance in plants.

Method/Technique	Species	Study/Experiment	References
Hyperaccumulation potential	*Urtica dioica* and *Sedum spectabile*	Plant specimens from uncontaminated sites were collected and transplanted in soils with Pb contamination without any additives, such as EDTA and HEDTA, and their natural hyper-tolerance and hyperaccumulation potentials were assessed through atomic spectroscopy.	[58]
Physiological indicators, morphological distribution, enrichment effects, and microstructure	*Koelreuteria paniculata*	Plants were exposed to various concentrations of Pb solution to explore impacts on growth, and transmission electron microscopy (TEM) and Fourier transform infrared spectroscopy (FTIR) were used to study physiology and microstructure.	[61]
Uptake and accumulation potential	*Pelargonium roseum*	Pb uptake and accumulation potential of the plant species was estimated using flame atomic absorption spectrophotometry (AAS).	[68]
Phytoremediation potential	*Codiaeum variegatum*	Phytoremediation of Pb-contaminated soil was studied by interpreting the phytoremediation potential of the plants through the following considerations: metal tolerance index (MTI), translocation factor (TF), and bioaccumulation factor (BAF).	[18]
Efficiency as a phytoremediator	*Crotalaria juncea*	The plant species was studied for its phytoremediation potential in Pb-contaminated soil, and metal concentrations in soil and plant samples were determined through atomic absorption spectrometry (AAS).	[36]
Effect on polyphenols, flavonoids, and proline contents	*Raphanus sativus* L.	The Pb stress indicators were determined using a UV-visible spectrophotometer.	[34]
Role as a potential phytoremediator	*Cordyline fruicosa* L.	Phytoremediation of Pb-contaminated soils was studied using a UV–visible spectrophotometer.	[37]
Effects on plant growth and development	*Raphanus raphanistrum*	Response of plant growth indicators was used as a novel strategy to indicate Pb contamination in soils around US schools before proceeding for testing.	[13]
Tolerance potential to elevated contents of Pb in organic and mineral soils	*Fagopyrum esculentum Moench*	A plant species was investigated for its tolerance in Pb-contaminated mineral and organic soils. Pb quantification in plant and soil samples was performed using atomic absorption spectrometry (AAS).	[30]

## Data Availability

There is no data availability statement.
